# Case-control study of craniomandibular disorders in patients with fibromyalgia

**DOI:** 10.4317/jced.51816

**Published:** 2015-04-01

**Authors:** Eduardo-José García-Moya, José-María Montiel-Company, José-Manuel Almerich-Silla

**Affiliations:** 1 Dentist, Healthcare Professional Training Teacher, Instituto Tierno Galván, Moncada, Valencia, Spain; 2 Assistant Lecturer, Department of Stomatology, University of Valencia, Spain; 3 Tenured Lecturer, Department of Stomatology, University of Valencia, Spain

## Abstract

**Background:**

Fibromyalgia is a clinical syndrome characterized by chronic widespread pain, which is non-articular and is predominantly experienced in the muscles and vertebral column, and by extensive heightened sensitivity to local pressure at many specific points The purpose of this study was to measure differences in the level of painful symptoms and in the movements of the mandible in a group of patients who had been diagnosed as suffering from fibromyalgia, in comparison with a control group. The anxiety and subjective pain levels and their relation with mandibular mobility were also compared.

**Material and Methods:**

A case-control study was designed. The temporomandibular joints and masticatory muscles of the cases (n=20) and controls (n=18) were examined, anxiety was assessed by the STAI index and subjective pain was measured on a visual analogue scale. The data analysis was carried out with SPSS v.19.0 software. The descriptive data were expressed as means and proportions at a 95% confidence interval. The proportions were compared with the chi-square test and the means with the Mann-Whitney U test. Pearson’s correlation coefficient was used to measure the association between quantitative variables.

**Results:**

The fibromyalgia patients (the case group) presented a higher level of pain following the musculoskeletal examination and significantly greater symptoms at the examination points. Regarding joint mobility, significant differences in mandibular opening were found (cases 43.4 mm vs controls 47.2 mm, p = 0.042). The mean pain score of the cases was significantly higher than that of the controls (4.03 vs 1.8, p = 0.001) but no significant differences were found in the anxiety index (23.8 vs 23.4).

**Conclusions:**

patients with fibromyalgia are affected to a greater extent by craniomandibular disorders, with lower mouth opening and higher pain levels than healthy persons. However, the anxiety levels of the two groups are similar.

** Key words:**Fibromyalgia, orofacial pain, temporomandibular disorder.

## Introduction

Fibromyalgia is a clinical syndrome characterized by chronic widespread pain, which is non-articular and is predominantly experienced in the muscles and vertebral column, and by extensive heightened sensitivity to local pressure at many specific points (1). Fatigue, sleep disorders and mood disorders are other frequent symptoms.

Several theories have been advanced to explain the physiopathology of this syndrome, including inflammatory or psychogenic causes, alpha-delta sleep, pain modulation disorder and serotonin deficit, among others. Fibromyalgia diagnosis is essentially clinical. Characteristically, the patients are women with long-standing widespread pain, fatigue, sleep disturbance and reproduci-ble trigger points.

In 1990 the American College of Rheumatology (2) published validated diagnostic criteria that continue to be used today:

• History of chronic widespread pain, present for longer than 3 months, that affects at least 3 of the 4 quadrants (the left and right sides of the body, above and below the waist) In addition, pain must be present in the axial skeleton (cervical spine, thoracic spine, lumbar spine or anterior thoracic wall).

• Pain on palpation of 11 of the 18 tender point sites (9 pairs of points): occipital bone insertion of the suboccipital muscles, ante-rior cervical aspect of the intertransverse spaces at c5-c7, midpoint of the upper borders of the trapezius, origin of the supraspinatus, second costochondral junction, 2 cm distal to the epicondyle, upper outer quadrant of the buttock, posterior surface of the greater trochanter, and the medial knee fat pad.

In the field of dentistry, craniomandibular disorders are an increasing problem. Numerous aetiological factors can contribute to their development (stress, anxiety, trauma, malocclusion, etc.) (3,4). Fibromyalgia and craniomandibular disorders share aetiological factors such as stress.

The high muscular pathology component in craniomandibular disorders and the muscular symptoms it shares with fibromyalgia suggested that the possible relations between the two conditions should be examined.

The main purpose of this study was to measure differences in the level of painful symptoms and in the lateral movements and opening of the mandible in a group of patients who had been diagnosed as suffering from fibromyalgia, in comparison with a control group. The anxiety and subjective pain levels and their relation with mandibular mobility in the two groups were also compared.

## Material and Methods

The study compared a case group (fibromyalgia sufferers) with a control group (healthy persons), following a case-control design. The study has been approved by a research ethics committee.

The sample size was 38, of whom 20 were cases and 18 controls. The cases were selected through a fibromyalgia association while the controls were patients from different dental practices.

The case selection criteria were as follows: women aged between 35 and 60 years, diagnosed with fibromyalgia, excluding diagnosis more than 5 years previously, and with no history of neck or orofacial pain, trauma or treatment prior to the fibromyalgia diagnosis. As a result of these exclusions, 4 were removed from the initial sample of 24 persons and the case group consequently numbered 20.

The control group was selected from dental patients with no known fibromyalgia or temporomandibular joint (TMJ) disorders, of the same gender (women) and age (35-60 years) as the case group, and finally numbered 18.

All the participants signed an informed consent form.

The basic temporomandibular disorder (TMD) screening questionnaire recommended by the American Academy of Orofacial Pain was self-administered to the sample. It asks 10 basic yes/no questions to detect a disorder.

1. Is it painful, difficult or both to open your mouth wide, for instance when you yawn?

2. Does your jaw sometimes lock or dislocate?

3. Is it painful, difficult or both to chew, talk or move your jaw?

4. Do you notice noises in your jaw joints?

5. Do you notice your jaw feeling stiff or tired?

6. Do you have pain in or around your ears, temples or cheeks?

7. Do you often have headaches or neck pain?

8. Have your recently suffered any injury to your head, neck or jaw?

9. Have you noticed any changes in your bite recently?

10. Have you ever been treated for jaw joint problems or facial or neck pain?

Both groups were then examined by a single examiner.

The physical examination included the following variables.

a) Measurement of the mandibular maximum passive mobility range, both in jaw opening and in excursions (lateral and protru-sion). Therabite® scales were used to measure the range of motion (5).

b) Palpation of the TMJ over the joint and in the retrodiscal zone. This procedure was mainly to detect tenderness on palpation, noting its presence or absence. The pain caused by examination of two anatomically different zones was quantified on the following scale: 0 (no pain), 1 (slight pain), 2 (moderate pain) or 3 (severe pain).

The examination of the retrodiscal or bilaminar zone was conducted to assess possible retrodiscitis.

Palpation over the joint was performed by locating the outermost part of the condyle and asking the patient to open and close his or her mouth several times.

c) Auscultation using a stethoscope and/or palpation for noises in the joint (clicking, crepitus).

d) Bilateral palpation of masseter and temporalis muscles. The purpose was to discover whether sensitivity to pain was present by palpating two predefined points on the masseter (superficial and deep) and three on the temporalis muscle (anterior, middle and posterior). Only one point on each muscle was used for result reporting.

• Masseter: pain in superficial part

• Temporalis: pain in anterior part.

e) Assessment of signs of excessive occlusal wear, loose teeth, tori, indentations on the tongue, or other signs of oral parafunction. This was effected through a general intra-oral examination using a mouth mirror and probe.

f) Visual analogical scale (VAS) Each patient was provided with a VAS-type scale on which to quantify the pain experienced in and around the joint before examination.

g) State-Trait Anxiety Inventory (STAI) questionnaire. The subjects answer each of the questions in the state anxiety part of the STAI questionnaire (S-anxiety) by scoring them on a four-point scale, numbered as follows: 0 (not at all), 1 (somewhat), 2 (moderately so) and 3 (very much so). In this questionnaire, each respondent’s score is calculated by adding up each of the columns and applying the weightings provided by the authors of the scale. The total ranges from 0 to 60. The cutoff point is 28 for men and 31 for women. The indices of internal consistency for the S-anxiety part of the Spanish version of this test are between 0.90 and 0.93. The reliability values calculated by the two-halves procedure reach 0.94.

The results were analysed using SPSS statistical software (v. 19.0). The descriptive data were expressed as means and proportions, with a 95% confidence interval. The chi-square test was employed for comparison of proportions and the Mann-Whitney U test for the means. Pearson’s correlation coefficient was used to measure the association between quantitative variables. The significance level was set at *p*<0.05.

## Results

On comparing the pain reported by cases and controls following the musculoskeletal examination, a higher percentage of individuals in the control group were free of pain at almost all the examination points.

The symptoms at the examination points on the masseter, palpation over the joint and palpation of the retrodiscal zone, signs, were significantly greater in the case group ( Table 1).

**Table 1:**
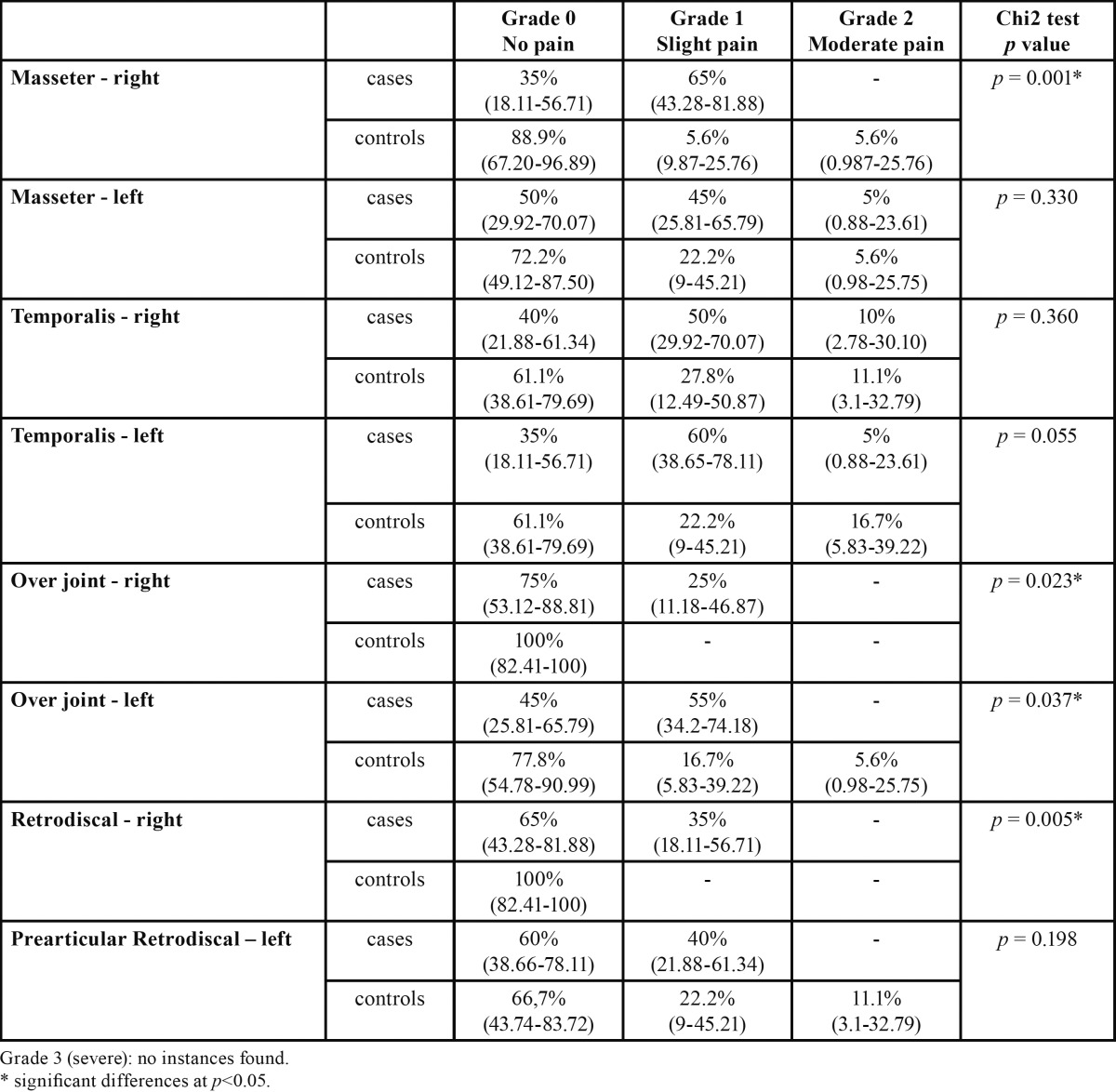
Musculoskeletal examination.

As regards clicking, the prevalence was very low (10-15%) and no significant differences were found between the two groups.

The jaw movement assessment ( Table 2) found significant differences in jaw opening, with the cases opening 43.4 mm and the controls 47.2 mm (Mann-Whitney U test, *p*=0.042), but no significant differences in left or right lateral movement. The mean VAS pain score for the cases was 4.03, significantly higher than the 1.80 of the controls (*p*=0.001), but no significant differences in anxiety levels were found between the two groups ( Table 2).

**Table 2:**
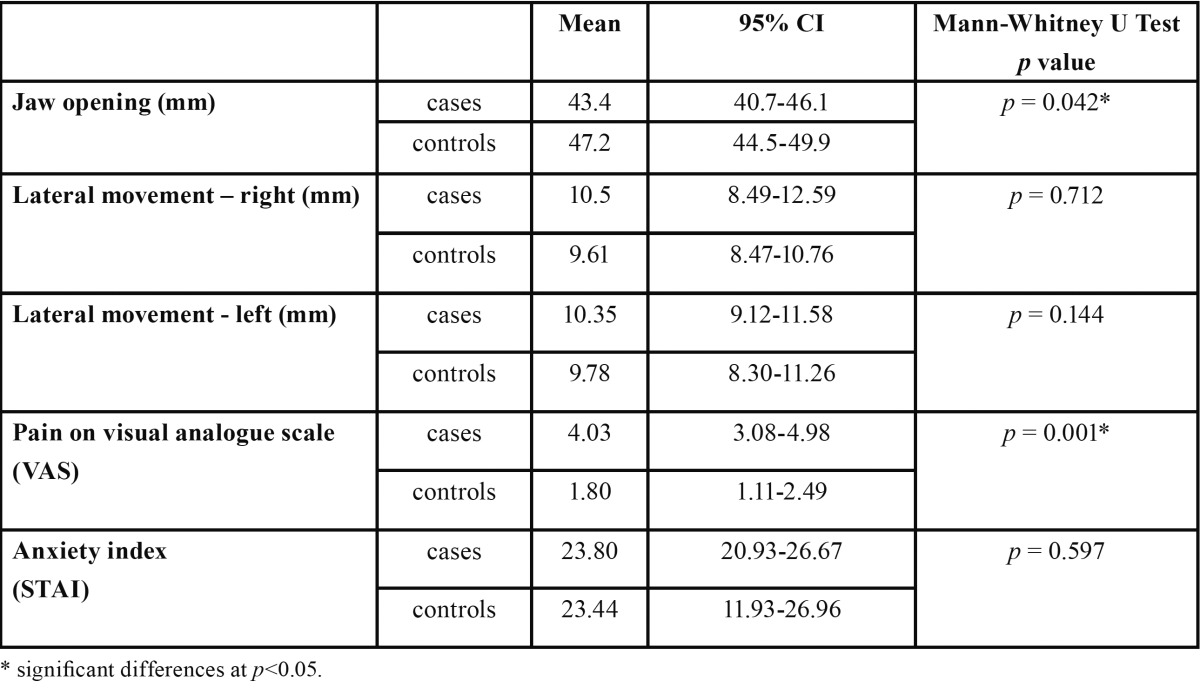
Comparison between cases and controls in jaw movement related variables and subjective pain (VAS) and anxiety (STAI) test scores.

 Table 3 shows a significant inverse linear correlation between jaw opening and VAS (Pearson’s correlation coefficient = -0.622) and between jaw opening and STAI (Pearson’s correlation coefficient = -0.458) in the case group. Moreover, VAS and STAI were also correlated (Pearson’s correlation coefficient = 0.496). In the control group, the only correlation was an inverse one between jaw opening and VAS (Pearson’s correlation coefficient = -0.670).

**Table 3:**
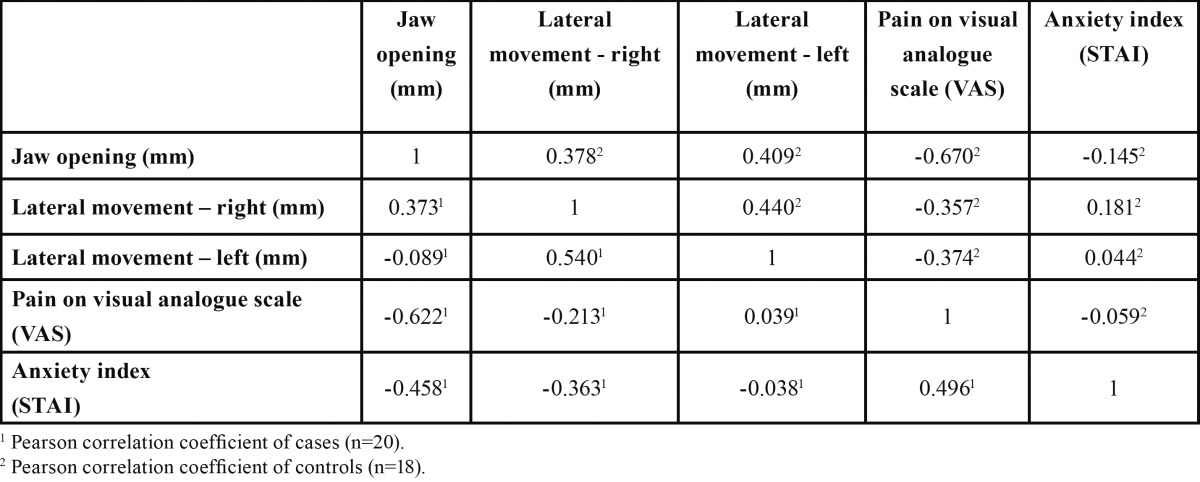
Pearson correlation coefficients for jaw movement related variables and subjective pain (VAS) and anxiety (STAI) test scores of cases and controls.

 Table 4 compares the percentages of each group’s responses to the AAOP questionnaire. In the following questions, the Yes ans-wers were significantly more numerous in the case group (chi square test *p*<0.05) than in the control group: pain or difficulty in opening the mouth (60% vs 22.2%), pain or difficulty in speaking or chewing (60% versus 22.2%), pain in the ears, temples or cheeks (95% vs 44%), frequent headaches or neck pain (100% vs 61.1%), recent changes in bite (40% vs 0%) and stiff or tired jaw (85% vs 33.3%). Of the fibromyalgia patients, 100% presented at least three affirmative answers to the questions, compared to 50% of the control group. The mean affirmative answer score for the cases was significantly higher than for the controls (5.8 vs 2.7, *p*= 0.00).

**Table 4:**
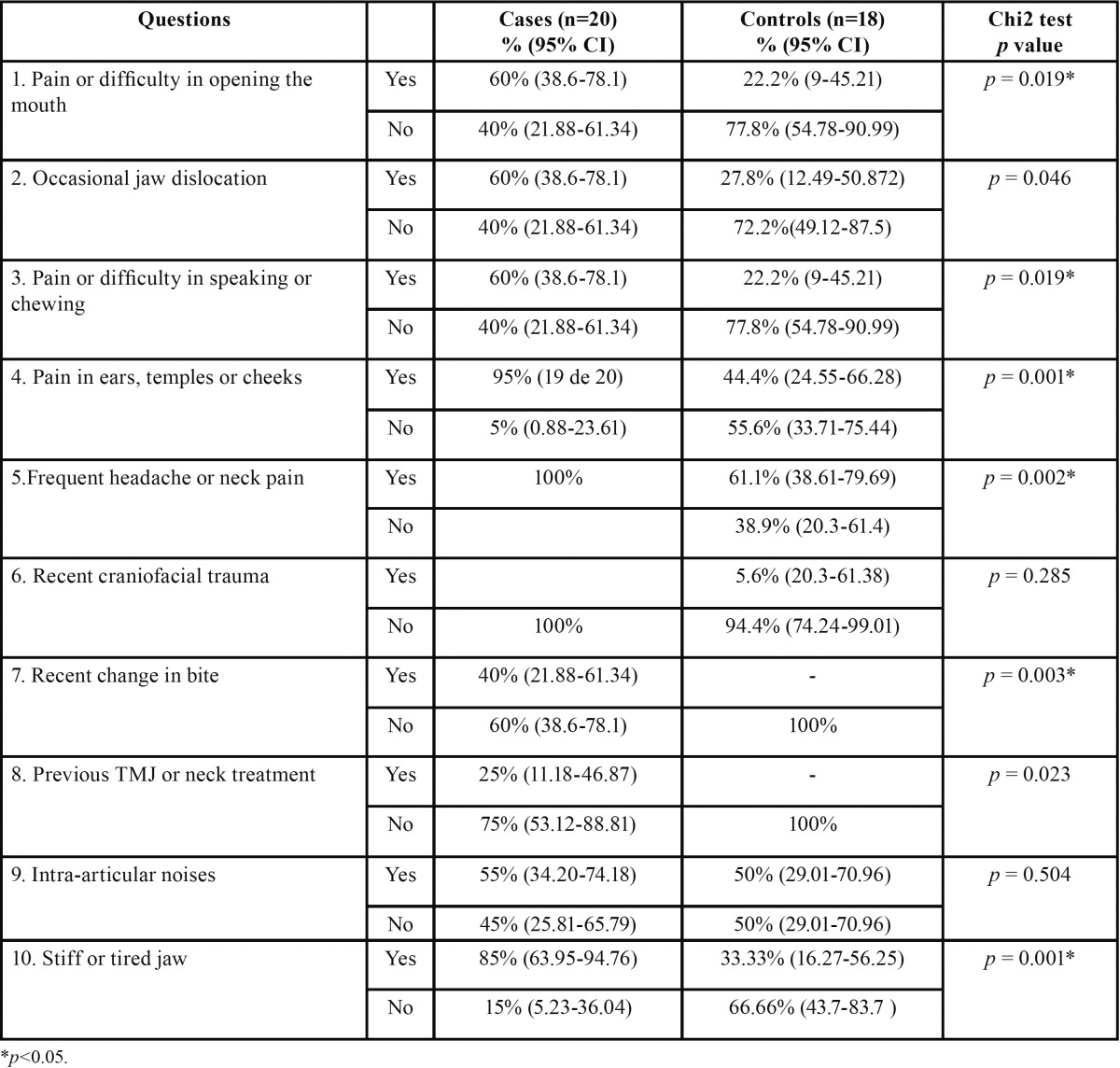
Comparison of percentage answers by cases and controls to the basic AAOP questionnaire.

## Discussion

The results of this study show that the patients diagnosed with fibromyalgia presented a significantly higher prevalence of signs and symptoms of craniomandibular disorders, such as reduced jaw opening, greater pain, painful muscle palpation at certain points, painful joint palpation and intra-articular noises.

The patients with fibromyalgia also gave more yes answers to the AAOP screening questionnaire.

Studies that relate craniomandibular disorders with fibromyalgia are few in number and use different methods (6-11).

Leblebici *et al.* (6) examined craniomandibular disorder prevalence in a group of women diagnosed with fibromyalgia and found that 80% presented TMJ disorders, mainly affecting the muscles. The present study found significant painful points on the masseter muscles in 65% of the case group.

Pleshy *et al.* (7) found that out of 60 patients diagnosed with fibromyalgia, 75% met the classification criteria for craniomandibular disorders of a muscular nature. Their data are similar to those of the present study and higher than those obtained by Korsun *et al.* (8), who detected some form of TMD in 42% of a group of 92 patients.

In 2007, Balasubramaniam *et al.* (1) assessed the TMJs of a group of women and men with fibromyalgia and found that 53% of the women but only 11% of the men met the diagnostic criteria for TMD. They concluded that hormonal disturbances might explain why the difference was so great. Given the particular characteristics of fibromyalgia, only women were included in the present study.

Both fibromyalgia and TMD have long been associated with a high number of concomitant disorders such as sleep disturbance, anxiety, stress, digestive problems, etc. It is worth noting that the STAI anxiety index did not prove significant in the fibromyalgia patients. The reason could be that the questionnaire used was the state anxiety part, which assesses the patient’s state at the moment of answering, rather than the trait anxiety part, which assesses anxiety in the past or over a long period of time. This could have led to a slight bias in the results.

Jaw aperture was significantly smaller in the cases than in the controls and the former experienced twice the pain of the latter. Jaw opening was significantly correlated with pain in both groups. Anxiety was only correlated with jaw opening in the case group. Korzum *et al.* has already shown the association between temporomandibular disorders and stress-related syndromes (8).

Another aspect to consider in these conditions is their possible shared aetiology (2,9). However, this is difficult to demonstrate as there is never one single factor that predisposes, generates or perpetuates these clinical manifestations, particularly in the TMJ. It would seem clear that both fibromyalgia and craniomandibular disorders present shared symptoms and signs, but it is highly debatable that they have a shared origin.

Aaron et al. (10) conducted a study to examine symptoms shared by fibromyalgia, craniomandibular disorders and chronic fatigue syndrome. They concluded that a series of disorders such as headache, irritable bowel syndrome or sensory alterations such as paraesthesia predominate in all three.

In a study by Hedenberg-Magnusson *et al.* (11), analysis of the questionnaires on craniomandibular disorders in patients with fibromyalgia showed that 94% of the sample presented symptoms of disorders. In the present study, 100% of the case group gave at least three affirmative answers to the questionnaire that the AAOP uses to screen for temporomandibular disorders.

Owing to the limited size of the study sample, these results need to be interpreted with caution. Studies of larger samples using longitudinal methods will be needed to establish reliably the relation between the two conditions.

## References

[1] Balasubramaniam R, Laudenbach JM, Stoopler ET (2007). Fibromyalgia: an update for oral health care providers. Oral Surg Oral Med Oral Pathol Oral Radiol Endod.

[2] Wolfe F, Smythe HA, Yunus MB, Bennett RM, Bombardier C, Goldenberg DL (1990). The American College of Rheumatology 1990 Criteria for the Classification of Fibromyalgia. Report of the Multicenter Criteria Committee. Arthritis Rheum.

[3] Murphy MK, Macbarb RF, Wong ME, Athanasiou KA (2013). Temporomandibular disorders: a review of etiology, clinical management, and tissue engineering strategies. Int J Oral Maxillofac Implants.

[4] Tanaka E, Detamore MS, Mercuri LG (2008). Degenerative disorders of the temporomandibular joint: etiology, diagnosis, and treatment. J Dent Res.

[5] Goulet JP, Clark GT, Flack VF, Liu C (1998). The reproducibility of muscle and joint tenderness detection methods and maximum mandibular movement measurement for the temporomandibular system. J Orofac Pain.

[6] Leblebici B, Pektaş ZO, Ortancil O, Hürcan EC, Bagis S, Akman MN (2007). Coexistence of fibromyalgia, temporomandibular disorder, and masticatory myofascial pain syndromes. Rheumatol Int.

[7] Plesh O, Wolfe F, Lane N (1996). The relationship between fibromyalgia and temporomandibular disorders: prevalence and symptom severity. J Rheumatol.

[8] Korszun A, Papadopoulos E, Demitrack M, Engleberg C, Crofford L (1998). The relationship between temporomandibular disorders and stress-associated syndromes. Oral Surg Oral Med Oral Pathol Oral Radiol Endod.

[9] Dao TT, Reynolds WJ, Tenenbaum HC (1997). Comorbidity between myofascial pain of the masticatory muscles and fibromyalgia. J Orofac Pain.

[10] Aaron LA, Burke MM, Buchwald D (2000). Overlapping conditions among patients with chronic fatigue syndrome, fibromyalgia, and temporomandibular disorder. Arch Intern Med.

[11] Hedenberg-Magnusson B, Ernberg M, Kopp S (1997). Symptoms and signs of temporomandibular disorders in patients with fibromyalgia and local myalgia of the temporomandibular system. A comparative study. Acta Odontol Scand.

